# The Institutional Library

**Published:** 1919-07-05

**Authors:** 


### THE INSTITUTIONAL LIBRARY.

**Dr. John Fothergill and His Friends.** By R. Hingston Fox, M.D. (London: Macmillan and Co., Ltd. 1919. Price 21s. net.)

The eighteenth century has fascinated many minds. Some have been drawn to its furniture and air of formal grace. Others to the hard intellectual qualities which are common to such different types as Hogarth, Pope, Johnson, and Lord Chesterfield. Others, again, delight in the sentiment of its china shepherdesses or Adam's interiors, and find in The Vicar of Wakefield and the Essays of Elia a grave simplicity which the world is supposed to have lost. Mr. Austin Dobson has become the poet of this tradition, and Sir James Barrie has set its sentiment upon the stage. But it is refreshing to turn from the great names in which the century still lives to a lesser, and for that reason a more typical, one; for Dr. John Fothergill represents the estimable professional man of the period when medicine was first giving its due value to clinical experience, and was no longer prepared to be bound by the tradition of the schools. Touching life at many points, Fothergill represents the best type of an educated and reflective mind at this point, and shows us better than a more outstanding figure could do the events of the time as they appeared to those observant of them. His interest in every branch of natural science, his collections of shells and fossils, the plants which he imported, the collectors whom he subsidised, brought Fothergill into contact with most of the intellect of his time in England and in America, and he was the friend and patron of numerous people. His generosity was really part of his culture, and, as his biographer well observes, "sometimes he would suggest a motive for his bounty that seemed to make the receiver a claimant and himself only discharging a debt." It was the same with his medical practice. He often refused fees, always from the clergy unless they were dignitaries, and to the end of his life part of his time was methodically devoted to attending the poor without charge. Sometimes he prescribed a banknote for his poorer patients, because hunger was a disease that could best be relieved by that means. He was really generous in that his generosity was not checked because it was sometimes abused.

An interesting chapter tells the story of the inoculation of the Empress Catharine II. of Russia by Dr. Dimsdale, who was recommended by Dr. Fothergill to the Russian Minister. The story of Dr. Dimsdale's journey, the secrecy with which the inoculation was performed, the arrangements made by the Empress for his flight in case it should prove unsuccessful, the honours with which he was eventually covered at her court, are fascinating reading. Few medical men have had such a patient or such rewards as the man who owed his chance to Dr. Fothergill.

A member of the Society of Friends, he supported the Quakers in their crusade against slavery, and John Howard in improving the condition of the gaols. He also, as early as 1754, favoured the public registration of births, marriages, and deaths. But it was not till 1801 that the first census was taken, and the Registration Act was not passed till 1837. As the life proceeds the interest widens, and finally we find Dr. Fothergill busy behind the scenes in affairs of State, the friend of Benjamin Franklin, and much occupied in the endeavour to prevent the separation of the American colonies which resulted from the war. His judgment, tact, and persistence were well displayed in this incursion into statesmanship, and he is only one of many examples of the truth that the very qualities necessary to a statesman are those which prevent his admission into politics, which are as corrupt to-day as ever. This is Franklin's tribute: "If we may estimate the goodness of a man by his disposition to do good, and his constant endeavour and success in doing it, I can hardly conceive that a better man has ever existed." Fothergill also took an active part in the affairs of the Society of Friends. He was a founder of Ackworth School, a model to the country, and the wonder is how he managed to combine so many activities with his scientific hobbies and considerable writings, both for learned societies, in the newspapers, and in frequent letters to his friends. His letter to a young lady before her marriage is a striking contrast to that written by Swift on a similar occasion, and we leave his life with admiration for the cultivated, agreeable, and generous spirit that it reveals. The professional man of action at his best, the doctor in general practice at his best, is here seen in a full-length portrait beside which are set those of many of his friends. The book is well documented, and has been carefully written by Dr. Hingston Fox, who has arranged his material with much skill, and preserved for us an inspiring personality.

**Prostitution in Europe.** By Abraham Flexner. Abridged Edition. (London: Grant Richards. 1919. Pp. 301. Price 6s. net.)

This treatise is one which should be thoroughly digested by every man and woman possessing the Parliamentary or municipal franchise, for it deals with a subject of enormous importance to the State, and one much neglected in this country until quite recently. That this counsel of perfection is unattainable in the present state of education and intelligence is undeniable; but at least it may be justly urged that no member of Parliament, Justice of the Peace, Poor-Law Guardian, member of a Watch Committee, clergyman, doctor, or police official should omit to study it. The one unfortunate point connected with this issue is due to the fact that the original was written in 1914 and has not been brought up to date, so that the influence of the war and of the ensuing armistice has not been taken into consideration. Mr. Flexner came to Europe in 1912 to study the problems of prostitution. He is a prominent educationalist in America, and he came with no orders except to ferret out truth, and to let no bias or preconceptions warp his judgment. It is this that makes his conclusions so exceptionally valuable, and his criticisms so illuminating. He is very scornful—and rightly—on the subject of the neglect in London to provide hospital accommodation, indoor and outdoor, for venereal disease: this refers to pre-war conditions, of course. So, too, his statement that England is indifferent on this subject—too true when it was written—is gradually becoming less true than it was. On the medical side of the subject there is but one slip: the statement (commonly believed up to a decade ago, but now discarded by medical opinion) that reinfections of syphilis do not take place. On general grounds this book is to be recommended to all who want to see their country a better place to live in, and their fellow-creatures happier and healthier.

**The Institutional Library**—(*continued*).

**Cerebro-Spinal Fever.** THE ETIOLOGY, SYMPTOMATOLOGY, DIAGNOSIS, AND TREATMENT OF EPIDEMIC CEREBRO-SPINAL MENINGITIS. By C. WORCESTER-DROUGHT, M.A., M.B. (Cantab.), Neurologist to the Royal Herbert Hospital, Woolwich; and ALEX. MILLS KENNEDY, M.D. (Glasgow), Bacteriologist to the Royal Herbert Hospital, Woolwich. (London: A. and C. Black, Ltd., Soho Square, W.)

THIS volume is the result of a happy combination between a practising neurologist and a professional bacteriologist, both of whom have had exceptional opportunities to study the disease as experienced during the war period. Much has already been published concerning the 1915 outbreak and the methods adopted to combat it, but in this work the authors have rendered the valuable service of collecting together all the major points, correlating them and illustrating the broad principles by numerous examples taken from their own fields of observation. Apparently all literature dealing with the disease has been consulted, the important sections given in very readable *précis*, and a relatively successful attempt made to bring the whole subject up to date. This book will undoubtedly prove of great worth to those who at any time may have to deal with the onset of cerebro-spinal fever either in epidemic or sporadic form, particularly under Service conditions; but at the same time there is much contained in its 485 pages which should be of the greatest interest to the civilian practitioner. The *pros* and *cons* of treatment are very fully discussed, and concise directions as to dosage, technique, and clinical indications provided. Eight full-page plates and fifty-six line illustrations and charts are published, giving considerable aid in making clear those practical points which, in words alone, it is always so hard to outline. On the whole, this unit of the Edinburgh Medicine Series leaves a most favourable memory with its readers, and impresses us as one of the completest volumes we have yet seen on an individual disease as seen during the past war.

**A Medical Field Service Handbook.** By C. MAX PAGE, D.S.O., M.S., F.R.C.S., Lieutenant-Colonel R.A.M.C. (S.R.), O.C. Field Ambulance. (London: Henry Frowde, Hodder and Stoughton. Oxford University Press. 1919. Price 6s. 6d.)

THE submarine campaign is responsible for more than direct loss of life and material; among other concomitant calamities, owing to the restricted supply of paper, it deprived army medical officers of that free interchange of experiences which would have resulted from their publication during the course of the war. Among other belated publications, the excellent manual by Colonel Max Page would have been invaluable earlier in the war. The first part deals with the causes of wastage, stress being laid upon the negligible proportion of those, such as typhoid, dysentery, and cholera, which in previous wars decimated armies. Then follows a detailed description of the long list of dirt diseases, which caused four-fifths of the wastage from disease on the Western Front; I.C.T., including scabies, impetigo, pyodermia, ulcers and boils caused two-fifths; trench fever, two-fifths, and infectious diseases and other ailments the remaining fifth. Thus body parasites were responsible for most of the disease wastage, the louse for trench fever, the acarus for scabies, and these, conjointly, for most of the other skin diseases. The organisation of sanitation at the front is sketched in general terms, including the means adopted for dealing with the louse and the acarus; that the ravages of these pests were inadequately contested is quite clear, and it is disappointing that this opportunity of urging reform was not seized. That lice-borne diseases can be entirely prevented has been demonstrated by the success of the methods adopted by the British Sanitary Commission to Serbia during the great epidemic of typhus in 1915, the applicability of the principle of which is fully discussed in an article in THE HOSPITAL (October 19, 1918), entitled "Two Disinfectors which Kill." The main requirements are a current steam of great volume under a pressure of 4-7 atmospheres, mobility by rail and by motor, the observance of a grouped system of disinfection, and baths, for the latter of which the French spray-bath described in THE HOSPITAL (July 13, 1918) is both most suitable and economical. The chapters on Trench Foot and Trench Fever are most lucid and interesting. A short chapter on trench nephritis on similar lines would have been a valuable addition, it is a disease of intense interest, which has been the subject of continual research in France.

By far the best chapters are those on the treatment of wounds, immediate and particular. The treatment of hæmorrhage and the prevention of shock and infection, the use of the tourniquet, the administration of morphia, the immobilisation of the various fractures, the use of extension and its pitfalls, the contra-indications for the transport of head injuries and the effects of toxic gases are included in the exhaustive list of problems which are explicitly solved for the inexperienced. The organisation of regimental aid posts and main dressing-stations are touched upon, but there is no description of the very useful "Rest Stations" for officers and for men, which are a so notable part of the activities of field ambulances. The literary style is good, the paper, printing, and binding are of pre-war excellence, and we confidently recommend this helpful guide to every junior army medical officer.

**A Text-Book of Sex Education for Teachers and Parents.** By WALTER M. GALLICHAN. (London: T. Werner Laurie. 6s.)

MR. WALTER GALLICHAN is the author of a book on the Psychology of Marriage which aims at removing the hindrances to happiness caused by ignorance of more than one kind. He puts forward now a *Text-Book of Sex Education* for parents and teachers which discusses not only the best means of shielding and enlightening the young, but the history, psychology, and bibliography of the subject. There is force in his contention that much mental and physical disease is traceable to uncomprehended sex impulses, terrors, false and hurtful revelations from unfit persons, or bad habits acquired in ignorance. All this has been said before, and Mr. Gallichan's volume does no more than collect the views of many writers and add practical suggestions for sets of lessons to young children and to older boys and girls, in which the aim is to combine enlightenment with reverence. There is no doubt that with the increasingly early freedom of both boys and girls, and especially with the growth of necessarily-unprotected professional life for the girl, the need of knowledge of the right care and use of the human body becomes imperative. If this book helps parents and teachers to do their duty in the matter, it will serve a good purpose. We cannot but feel that if Mr. Stephen Paget's idea obtains currency, and civilised society again welcomes the adolescent with ceremony, as primitive peoples do, though without their usually horrible rites, the task will become progressively easier. Mr. Gallichan has some wise things to say upon the influences of school, of social standards and amusements, and of books read upon the developing natures of the young. Perhaps the most widely useful thought which he suggests is that not even those least concerned with any form of education can hold themselves without responsibility for the influences which elevate or corrupt these citizens in the making.

